# Crystal structure and Hirshfeld surface analysis of (*E*)-3-(2-chloro-4-fluoro­phen­yl)-1-(2,5-di­chloro­thio­phen-3-yl)prop-2-en-1-one

**DOI:** 10.1107/S2056989018010216

**Published:** 2018-07-24

**Authors:** T. N. Sanjeeva Murthy, S. Naveen, C. S. Chidan Kumar, M. K. Veeraiah, Ching Kheng Quah, B. P. Siddaraju, Ismail Warad

**Affiliations:** a Department of Chemistry, Sri Siddhartha Academy of Higher Education, Tumkur 572 107, Karnataka, India; b Department of Physics, School of Engineering and Technology, Jain University, Bangalore 562 112, India; c Department of Engineering Chemistry, Vidya Vikas Institute of Engineering & Technology, Visvesvaraya Technological University, Alanahally, Mysuru 570 028, Karnataka, India; d Department of Chemistry, Sri Siddhartha Institute of Technology, Tumkur 572 105, Karnataka, India; e X-ray Crystallography Unit, School of Physics, Universiti Sains Malaysia, 11800 USM, Penang, Malaysia; f Department of Chemistry, Cauvery Institute of Technology, Mandya 571 402, Karnataka, India; g Department of Chemistry, Science College, An-Najah National University, PO Box 7, Nablus, West Bank, Palestinian Territories

**Keywords:** Thio­phene chalcone, crystal structure, R_{2}^{2}(8) ring motif, hydrogen bonding, Hirshfeld surface analysis

## Abstract

In the title chalcone–thio­phene derivative, C_13_H_6_Cl_3_FOS, the aromatic rings are inclined to one another by 12.9 (2)°, and the thio­phene ring is affected by π-conjugation. In the crystal, mol­ecules are linked by C—H⋯F hydrogen bonds, forming an *R*_2_^2^(8) ring motif. A Hirshfeld surface analysis was conducted to verify the contribution of the different inter­molecular inter­actions. The shape-index surface clearly shows that the two sides of the mol­ecules are involved in the same contacts with neighbouring mol­ecules and the curvedness plots show flat surface patches characteristic of planar stacking.

## Abstract 2

In the crystal, the mol­ecules are linked by weak C–H⋯F hydrogen bonds into the supra­molecular inversion dimers.

## Chemical context   

Natural products are important sources in the search for new agents for cancer therapies with minimal side effects. Chalcones, considered to be the precursor of flavonoids and isoflavonoids, are abundant in edible plants. Compounds with the 1,3-di­phenyl­prop-2-en-1-one framework are described by its generic term ‘chalcone’. They consist of open-chain flavonoids in which the two aromatic rings are joined by a three-carbon α,β-unsaturated carbonyl system. These are coloured compounds because of the presence of the –CO—CH=CH– chromophore, which depends in the presence of other auxochromes. Accumulating evidence has shown that chalcones and their derivatives could inhibit tumor initiation and progression. In view of the above, and as a part of our ongoing research on chalcone derivatives (Naveen *et al.*, 2017 ▸; Lokeshwari *et al.*, 2017 ▸; Tejkiran *et al.*, 2016 ▸), we report herein the synthesis, crystal structure and Hirshfeld surface analysis of the title compound.
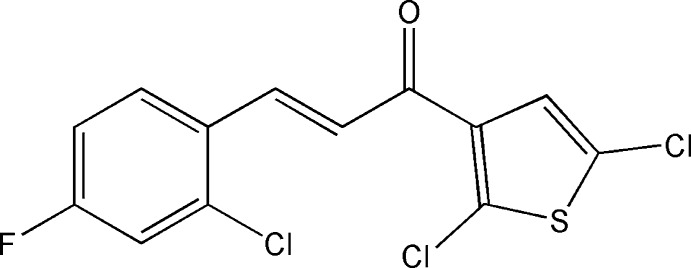


## Structural commentary   

**Figure 1:**
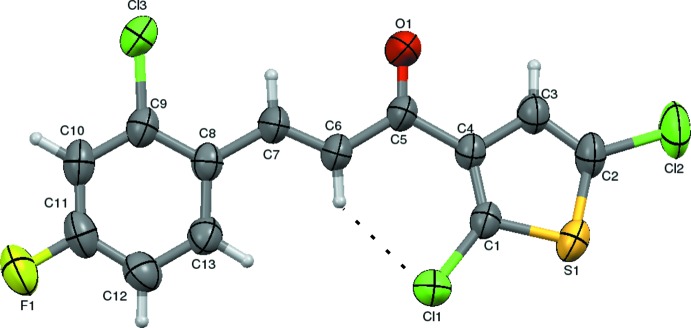
The mol­ecular structure of the title compound, indicating the atom-numbering scheme. The intra­molecular C—H⋯Cl hydrogen bond (dashed line) closes an *S*(6) motif. Displacement ellipsoids are drawn at the 50% probability level.

The mol­ecular structure of the title compound, shown in Fig. 1 ▸, is comprised of two aromatic rings (chloro­fluoro­phenyl and di­chloro­thio­phene) linked by C=C—C(=O)—C enone bridge. The bond lengths and bond angles are normal and the mol­ecular conformation is characterized by a dihedral angle of 12.9 (2)° between the mean planes of the two aromatic rings. The olefinic double bond C6=C7 of 1.303 (6) Å is in an *E* configuration and is C*sp* ^2^ hybridized. The unsaturated keto group is in a syn-periplanar conformation with respect to the olefenic double bond, which is evident from the torsion angle value of −0.5 (8)° for the atoms O1—C5—C6—C7. The thio­phene ring is affected by π conjugation. This can be explained by the longer C=S values of 1.703 (6) and 1.714 (4) Å for S1=C2 and S1=C1, respectively. The bond-angle values O1—C5—C6 [121.9 (4)°], O1—C5—C4 [118.2 (4)°] and C5—C6—C7 = 125.14 (4)° about C5 indicate that the carbon atom is in a distorted trigonal–planar configuration, which is due to steric hindrance of the oxygen atom. The mol­ecular structure is stabilized by an intra­molecular C6—-H6*A*⋯Cl1 hydrogen bond (Table 1 ▸) that closes an *S*(6) motif, as shown in Fig. 1 ▸.

## supra­molecular features   

**Figure 2:**
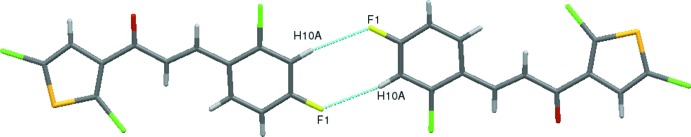
The 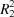
(8) ring motif.

In the crystal, the mol­ecules are linked by C—H⋯F hydrogen bonds, forming an 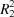
(8) ring motif as shown in Fig. 2 ▸. The structure also features π–π inter­actions: *Cg*1⋯*Cg*1(*x* − 1, *y*, *z*) = 3.956 (3) Å [α = 0°, β = 24.0°, γ = 24.0°, perpendicular distance of *Cg*1 on itself = 3.6131 (19) Å] and *Cg*2⋯*Cg*2(*x* + 1, *y*, *z*) = 3.957 (3) Å [α = 0°, β = 27.3°, γ = 27.3°] where *Cg*1 and *Cg*2 are the centroids of the S1/C1–C4 and C8–C13 rings, respectively.

## Database survey   

A survey of the Cambridge Structural Database (CSD, Version 5.39, last update November 2016; Groom *et al.*, 2016 ▸) using (*E*)-3-(phen­yl)-1-(2,5-di­chloro­thio­phen-3-yl)prop-2-en-1-one as the main skeleton revealed the presence of three structures containing a similar 2,5-di­chloro­thio­phene–chalcone moiety to the title compound but with different substituents on the terminal phenyl rings, *viz*. [(*E*)-1-(2,5-di­chloro-3-thien­yl)-3-(*X*)prop-2-en-1-one], where *X* = 4-(di­methyl­amino)­phenyl (Dutkiewicz *et al.*, 2010 ▸), 3,4-di­meth­oxy­phenyl (Harrison *et al.*, 2010*a* ▸) and 6-meth­oxy-2-naphthyl (Jasinski *et al.*, 2010 ▸). In these three compounds, the dihedral angles between the central and terminal phen­yl/naphthyl ring are in the range 2.13–11.90°. The difference may arise from the inter­molecular hydrogen bonds between adjacent mol­ecules.

## Hirshfeld surface analysis   

**Figure 3:**
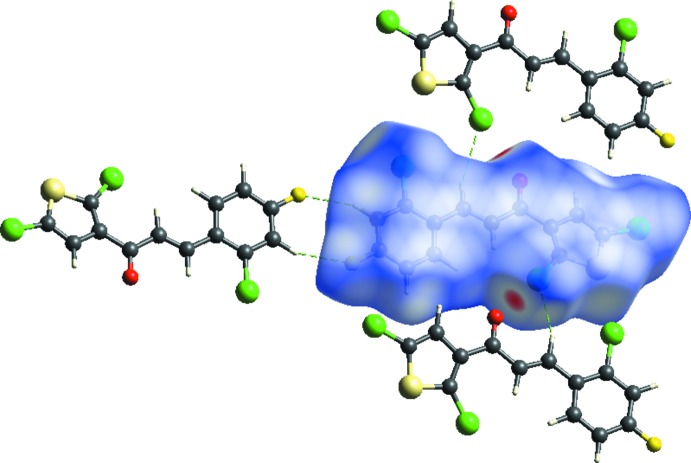
View of the three-dimensional Hirshfeld surface of the title compound mapped over *d*_norm_.

Hirshfeld surfaces and fingerprint plots were generated for the title compound based on the crystallographic information file (CIF) using *CrystalExplorer* (McKinnon *et al.*, 2007 ▸). Hirshfeld surfaces enable the visualization of inter­molecular inter­actions with different colours and colour intensity representing short or long contacts and indicating the relative strength of the inter­actions. Figs. 3 ▸ and 4 ▸ show the Hirshfeld surfaces mapped over *d*_norm_ (−0.139 to 1.120 a.u.) and shape-index (−1.0 to 1.0 a.u.), respectively. The calculated volume inside the Hirshfeld surface is 325.37 Å^3^ in the area of 310.17 Å^3^.

**Figure 4:**
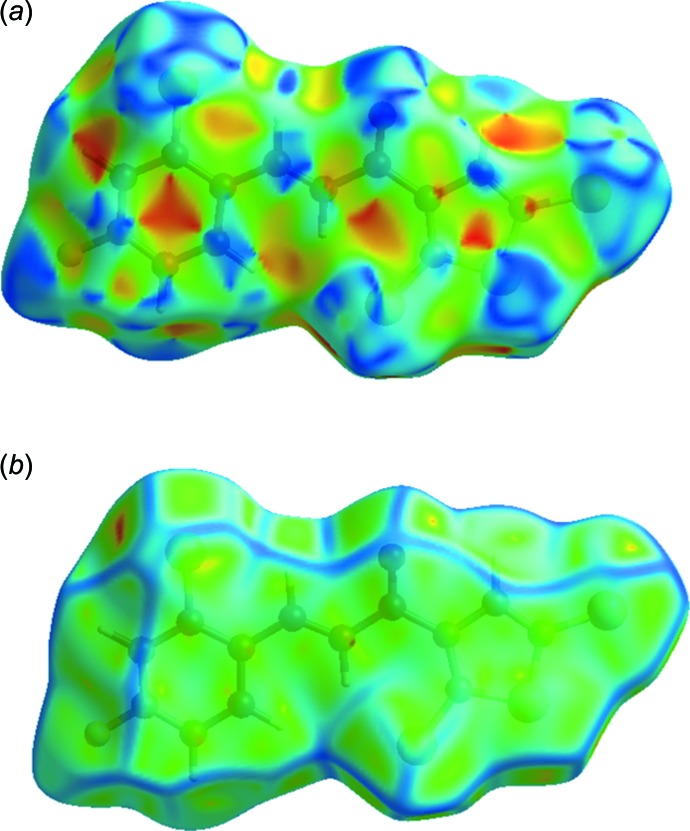
Hirshfeld surface of the title compound mapped over (*a*) shape-index and (*b*) curvedness.

In Fig. 4 ▸, the dark spots near atoms Cl1 and F1 result from the C6—H6*A*⋯Cl1 and C10—H10*A*⋯F1 inter­actions, which play a significant role in the mol­ecular packing of the title compound. The Hirshfeld surfaces illustrated in Fig. 4 ▸ also reflect the involvement of different atoms in the inter­molecular inter­actions through the appearance of blue and red regions around the participating atoms, which correspond to positive and negative electrostatic potential, respectively. The shape-index surface clearly shows that the two sides of the mol­ecules are involved in the same contacts with neighbouring mol­ecules while the curvedness plots show flat surface patches characteristic of planar stacking.

**Figure 5:**
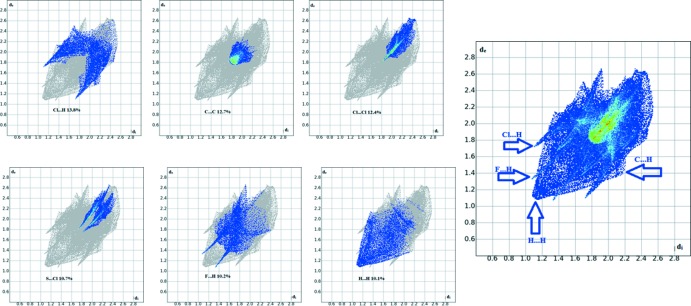
Two-dimensional fingerprint plots showing the percentage contributions of the various inter­actions.

The overall two-dimensional fingerprint plot for the title compound and those delineated into Cl⋯H/H⋯Cl, C⋯C, Cl⋯Cl, Cl⋯S/S⋯Cl, H⋯H, F⋯H/H⋯F, C⋯H/H⋯C contacts are illustrated in Fig. 5 ▸; the percentage contributions from the different inter­atomic contacts to the Hirshfeld surfaces are as follows: Cl⋯H (13.8%), C⋯C (12.7%), Cl⋯Cl (12.4%), Cl⋯S (10.7%), F⋯H (10.2%), H⋯H (10.1%), C⋯H (8.3%). The percentage contributions for other inter­molecular contacts are less than 5% in the Hirshfeld surface mapping.

## Synthesis and crystallization   

**Figure 6:**
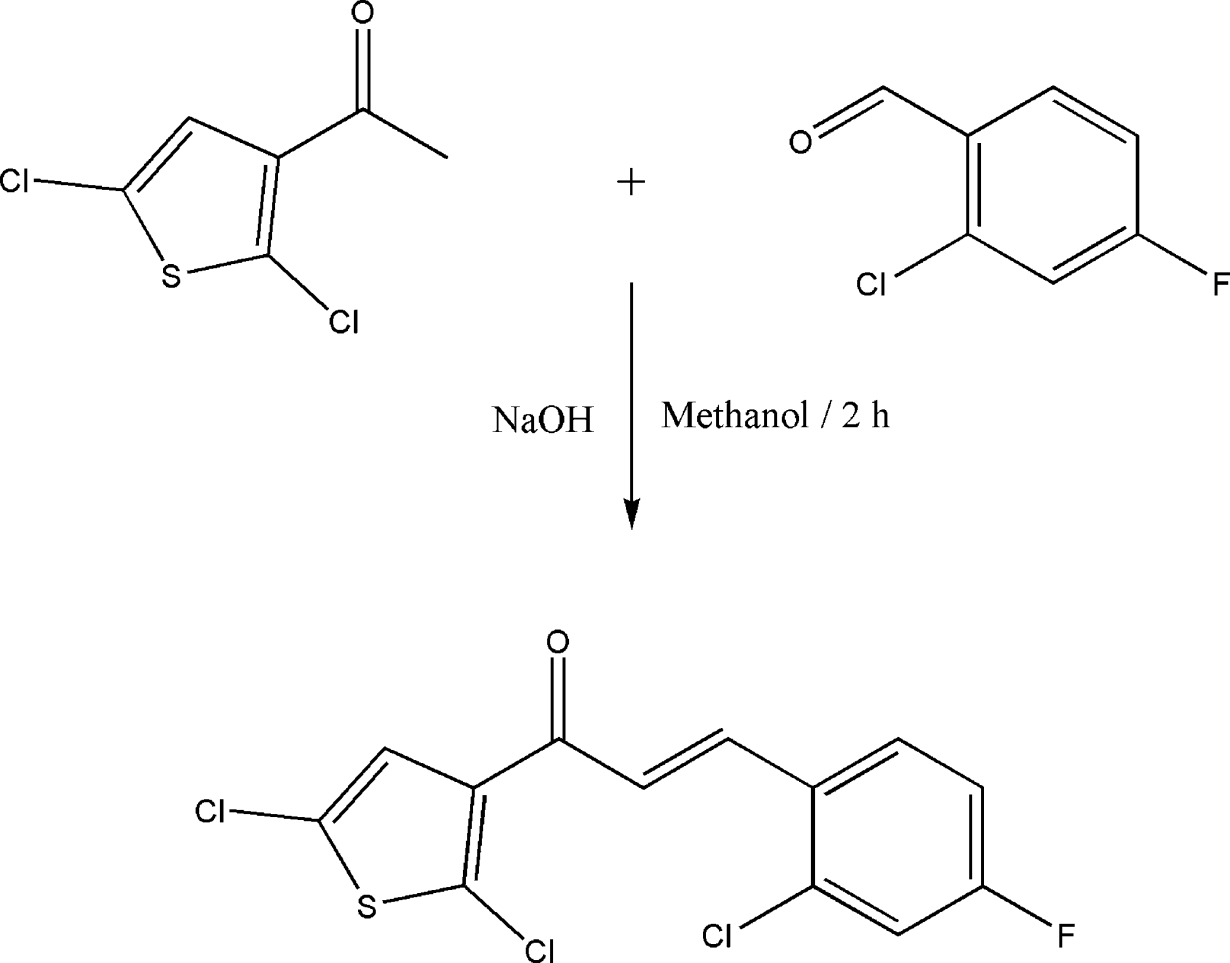
Synthesis of the title compound.

The title compound was synthesized as per the procedure reported earlier (Kumar *et al.*, 2013*a* ▸,*b* ▸; Chidan Kumar *et al.*, 2014 ▸). 1-(2,5-Di­chloro­thio­phen-3-yl)ethanone (0.01 mol) (Harrison *et al.*, 2010*b* ▸) and 2,4-di­chloro­benzaldehyde (0.01 mol) were dissolved in 20 ml of methanol. A catalytic amount of NaOH was added to the solution dropwise with vigorous stirring. The reaction mixture was stirred for about 2 h at room temperature. The formed crude products were filtered off, washed successively with distilled water and recrystallized from methanol to give the title chalcone. The reaction scheme is shown in Fig. 6 ▸. The melting point (306–309 K) was determined using a Stuart Scientific (UK) apparatus.

## Refinement   

Crystal data, data collection and structure refinement details are summarized in Table 2 ▸. C-bound H atoms were positioned geometrically (C—H = 0.95–0.99 Å) and refined using a riding model with *U*_iso_(H) = 1.2*U*_eq_(C).

## Supplementary Material

Crystal structure: contains datablock(s) global, I. DOI: 10.1107/S2056989018010216/xu5930sup1.cif

Structure factors: contains datablock(s) I. DOI: 10.1107/S2056989018010216/xu5930Isup2.hkl

CCDC reference: 1036795

Additional supporting information:  crystallographic information; 3D view; checkCIF report

## Figures and Tables

**Table 1:** Hydrogen-bond geometry (Å, °)

*D*—H⋯*A*	*D*—H	H⋯*A*	*D*⋯*A*	*D*—H⋯*A*
C6—H6*A*⋯Cl1	0.93	2.47	3.207 (5)	136
C10—H10*A*⋯F1^i^	0.93	2.54	3.433 (6)	160

Symmetry code: (i) 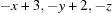.

**Table 2:** Experimental details

Crystal data	
Chemical formula	C_13_H_6_Cl_3_FOS
*M*_r_	335.60
Crystal system, space group	Monoclinic, *P*2_1_/*c*
Temperature (K)	294
*a*, *b*, *c* (Å)	3.9564 (8), 13.367 (2), 25.173 (5)
β (°)	93.363 (4)
*V* (Å^3^)	1329.0 (4)
*Z*	4
Radiation type	Mo *K*α
μ (mm^−1^)	0.84
Crystal size (mm)	0.44 × 0.19 × 0.14

Data collection	
Diffractometer	Bruker APEXII DUO CCD area-detector
Absorption correction	Multi-scan (*SADABS*; Bruker, 2012 ▸)
*T*_min_, *T*_max_	0.708, 0.894
No. of measured, independent and observed [*I* > 2σ(*I*)] reflections	3901, 3901, 2430
*R*_int_	0.000
(sin θ/λ)_max_ (Å^−1^)	0.707

Refinement	
*R*[*F* ^2^ > 2σ(*F* ^2^)], *wR*(*F* ^2^), *S*	0.076, 0.218, 1.04
No. of reflections	3901
No. of parameters	173
H-atom treatment	H-atom parameters constrained
Δρ_max_, Δρ_min_ (e Å^−3^)	0.46, −0.48

Computer programs: *APEX2* and *SAINT* (Bruker, 2012 ▸), *SHELXS97* (Sheldrick, 2008 ▸), *SHELXL2013* (Sheldrick, 2015 ▸), *Mercury* (Macrae *et al.*, 2006 ▸) and *PLATON* (Spek, 2009 ▸).

## supplementary crystallographic information

### Crystal data

**Table:**

C_13_H_6_Cl_3_FOS	*F*(000) = 672
*M**_r_* = 335.60	*D*_x_ = 1.677 Mg m^−^^3^
Monoclinic, *P*2_1_/*c*	Mo *K*α radiation, λ = 0.71073 Å
Hall symbol: -P 2ybc	Cell parameters from 2430 reflections
*a* = 3.9564 (8) Å	θ = 1.6–30.2°
*b* = 13.367 (2) Å	µ = 0.84 mm^−^^1^
*c* = 25.173 (5) Å	*T* = 294 K
β = 93.363 (4)°	Rectangle, green
*V* = 1329.0 (4) Å^3^	0.44 × 0.19 × 0.14 mm
*Z* = 4	

### Data collection

**Table:**

Bruker APEXII DUO CCD area-detector diffractometer	3901 independent reflections
Radiation source: Rotating Anode	2430 reflections with *I* > 2σ(*I*)
Graphite monochromator	*R*_int_ = 0.0000
Detector resolution: 18.4 pixels mm^-1^	θ_max_ = 30.2°, θ_min_ = 1.6°
φ and ω scans	*h* = −5→5
Absorption correction: multi-scan (SADABS; Bruker, 2012)	*k* = −18→18
*T*_min_ = 0.708, *T*_max_ = 0.894	*l* = −2→35
3901 measured reflections	

### Refinement

**Table:**

Refinement on *F*^2^	Primary atom site location: structure-invariant direct methods
Least-squares matrix: full	Secondary atom site location: difference Fourier map
*R*[*F*^2^ > 2σ(*F*^2^)] = 0.076	Hydrogen site location: inferred from neighbouring sites
*wR*(*F*^2^) = 0.218	H-atom parameters constrained
*S* = 1.04	*w* = 1/[σ^2^(*F*_o_^2^) + (0.0673*P*)^2^ + 2.8657*P*] where *P* = (*F*_o_^2^ + 2*F*_c_^2^)/3
3901 reflections	(Δ/σ)_max_ < 0.001
173 parameters	Δρ_max_ = 0.46 e Å^−^^3^
0 restraints	Δρ_min_ = −0.48 e Å^−^^3^

### Special details

**Table:**

Geometry. Bond distances, angles etc. have been calculated using the rounded fractional coordinates. All su's are estimated from the variances of the (full) variance-covariance matrix. The cell esds are taken into account in the estimation of distances, angles and torsion angles
Refinement. Refinement on F^2^ for ALL reflections except those flagged by the user for potential systematic errors. Weighted R-factors wR and all goodnesses of fit S are based on F^2^, conventional R-factors R are based on F, with F set to zero for negative F^2^. The observed criterion of F^2^ > 2sigma(F^2^) is used only for calculating -R-factor-obs etc. and is not relevant to the choice of reflections for refinement. R-factors based on F^2^ are statistically about twice as large as those based on F, and R-factors based on ALL data will be even larger.

### Fractional atomic coordinates and isotropic or equivalent isotropic displacement parameters (Å^2^)

**Table:**

	*x*	*y*	*z*	*U*_iso_*/*U*_eq_	
Cl1	0.3576 (4)	0.89705 (9)	0.29850 (5)	0.0644 (5)	
Cl2	0.2067 (5)	1.14124 (14)	0.48102 (5)	0.0892 (6)	
Cl3	1.1255 (5)	1.22982 (11)	0.10663 (6)	0.0863 (6)	
S1	0.2273 (3)	0.97600 (10)	0.40296 (5)	0.0581 (4)	
F1	1.3011 (10)	0.8888 (3)	0.03284 (14)	0.0890 (16)	
O1	0.6247 (13)	1.2332 (3)	0.28547 (15)	0.0826 (16)	
C1	0.3669 (11)	0.9974 (3)	0.34077 (16)	0.0452 (11)	
C2	0.2951 (13)	1.0983 (4)	0.41922 (17)	0.0576 (15)	
C3	0.4177 (12)	1.1522 (4)	0.37994 (16)	0.0518 (16)	
C4	0.4643 (11)	1.0936 (3)	0.33320 (15)	0.0438 (11)	
C5	0.6036 (13)	1.1423 (3)	0.28592 (16)	0.0503 (14)	
C6	0.7148 (13)	1.0813 (3)	0.24247 (17)	0.0528 (14)	
C7	0.8427 (14)	1.1157 (4)	0.19955 (16)	0.0553 (14)	
C8	0.9626 (11)	1.0570 (3)	0.15588 (15)	0.0446 (11)	
C9	1.0932 (12)	1.1014 (4)	0.11131 (17)	0.0505 (16)	
C10	1.2095 (12)	1.0457 (4)	0.06983 (17)	0.0567 (16)	
C11	1.1865 (13)	0.9448 (4)	0.0731 (2)	0.0621 (19)	
C12	1.0579 (14)	0.8963 (4)	0.1151 (2)	0.0633 (17)	
C13	0.9467 (13)	0.9528 (4)	0.15625 (18)	0.0535 (16)	
H3A	0.46730	1.22000	0.38260	0.0620*	
H6A	0.69310	1.01230	0.24550	0.0630*	
H7A	0.85890	1.18480	0.19660	0.0660*	
H10A	1.30020	1.07640	0.04070	0.0680*	
H12A	1.04550	0.82680	0.11580	0.0760*	
H13A	0.85860	0.92070	0.18510	0.0640*	

### Atomic displacement parameters (Å^2^)

**Table:**

	*U*^11^	*U*^22^	*U*^33^	*U*^12^	*U*^13^	*U*^23^
Cl1	0.0855 (10)	0.0491 (6)	0.0602 (7)	−0.0030 (6)	0.0169 (6)	−0.0015 (5)
Cl2	0.1049 (13)	0.1212 (13)	0.0442 (6)	−0.0094 (11)	0.0269 (7)	−0.0147 (7)
Cl3	0.1298 (15)	0.0642 (8)	0.0694 (8)	−0.0049 (9)	0.0430 (9)	0.0128 (6)
S1	0.0607 (8)	0.0698 (8)	0.0448 (6)	−0.0031 (6)	0.0114 (5)	0.0138 (5)
F1	0.098 (3)	0.096 (3)	0.076 (2)	0.013 (2)	0.030 (2)	−0.0231 (18)
O1	0.141 (4)	0.0503 (19)	0.060 (2)	−0.011 (2)	0.037 (2)	−0.0004 (16)
C1	0.043 (2)	0.054 (2)	0.0392 (18)	0.0025 (19)	0.0072 (16)	0.0064 (16)
C2	0.059 (3)	0.078 (3)	0.0365 (19)	−0.005 (3)	0.0095 (19)	−0.004 (2)
C3	0.056 (3)	0.060 (3)	0.040 (2)	−0.003 (2)	0.0078 (19)	−0.0031 (18)
C4	0.045 (2)	0.051 (2)	0.0357 (18)	−0.0023 (19)	0.0060 (16)	0.0001 (16)
C5	0.066 (3)	0.048 (2)	0.0378 (19)	−0.004 (2)	0.0118 (19)	0.0039 (17)
C6	0.067 (3)	0.051 (2)	0.042 (2)	0.000 (2)	0.017 (2)	0.0039 (18)
C7	0.076 (3)	0.052 (2)	0.039 (2)	0.000 (2)	0.013 (2)	0.0023 (17)
C8	0.041 (2)	0.056 (2)	0.0367 (18)	0.0005 (19)	0.0016 (16)	0.0010 (16)
C9	0.050 (3)	0.061 (3)	0.041 (2)	0.000 (2)	0.0061 (18)	0.0071 (18)
C10	0.050 (3)	0.081 (3)	0.040 (2)	0.002 (3)	0.0093 (19)	0.001 (2)
C11	0.052 (3)	0.081 (4)	0.054 (3)	0.009 (3)	0.010 (2)	−0.015 (2)
C12	0.061 (3)	0.059 (3)	0.071 (3)	0.010 (3)	0.013 (3)	−0.006 (2)
C13	0.057 (3)	0.057 (3)	0.047 (2)	0.006 (2)	0.008 (2)	0.0071 (19)

### Geometric parameters (Å, º)

**Table:**

Cl1—C1	1.711 (4)	C7—C8	1.453 (6)
Cl2—C2	1.713 (5)	C8—C9	1.395 (6)
Cl3—C9	1.726 (6)	C8—C13	1.394 (7)
S1—C1	1.714 (4)	C9—C10	1.383 (7)
S1—C2	1.703 (5)	C10—C11	1.355 (8)
F1—C11	1.359 (6)	C11—C12	1.364 (7)
O1—C5	1.218 (6)	C12—C13	1.375 (7)
C1—C4	1.359 (6)	C3—H3A	0.9300
C2—C3	1.337 (7)	C6—H6A	0.9300
C3—C4	1.434 (6)	C7—H7A	0.9300
C4—C5	1.490 (6)	C10—H10A	0.9300
C5—C6	1.453 (6)	C12—H12A	0.9300
C6—C7	1.303 (6)	C13—H13A	0.9300
			
C1—S1—C2	90.3 (2)	Cl3—C9—C10	117.0 (4)
Cl1—C1—S1	116.2 (2)	C8—C9—C10	122.2 (5)
Cl1—C1—C4	130.6 (3)	C9—C10—C11	117.6 (4)
S1—C1—C4	113.3 (3)	F1—C11—C10	118.5 (4)
Cl2—C2—S1	120.2 (3)	F1—C11—C12	118.2 (5)
Cl2—C2—C3	126.4 (4)	C10—C11—C12	123.4 (5)
S1—C2—C3	113.5 (4)	C11—C12—C13	118.3 (5)
C2—C3—C4	112.5 (5)	C8—C13—C12	121.8 (4)
C1—C4—C3	110.5 (4)	C2—C3—H3A	124.00
C1—C4—C5	130.3 (4)	C4—C3—H3A	124.00
C3—C4—C5	119.2 (4)	C5—C6—H6A	117.00
O1—C5—C4	118.2 (4)	C7—C6—H6A	117.00
O1—C5—C6	121.9 (4)	C6—C7—H7A	117.00
C4—C5—C6	119.9 (4)	C8—C7—H7A	117.00
C5—C6—C7	125.1 (4)	C9—C10—H10A	121.00
C6—C7—C8	126.6 (5)	C11—C10—H10A	121.00
C7—C8—C9	122.1 (4)	C11—C12—H12A	121.00
C7—C8—C13	121.2 (4)	C13—C12—H12A	121.00
C9—C8—C13	116.7 (4)	C8—C13—H13A	119.00
Cl3—C9—C8	120.7 (4)	C12—C13—H13A	119.00
			
C2—S1—C1—Cl1	−178.6 (3)	C4—C5—C6—C7	−179.4 (5)
C2—S1—C1—C4	0.7 (4)	C5—C6—C7—C8	178.7 (5)
C1—S1—C2—Cl2	179.7 (3)	C6—C7—C8—C9	179.5 (5)
C1—S1—C2—C3	−0.3 (4)	C6—C7—C8—C13	0.6 (8)
Cl1—C1—C4—C3	178.2 (4)	C7—C8—C9—Cl3	1.2 (6)
Cl1—C1—C4—C5	−1.8 (8)	C7—C8—C9—C10	179.6 (5)
S1—C1—C4—C3	−0.9 (5)	C13—C8—C9—Cl3	−179.9 (4)
S1—C1—C4—C5	179.0 (4)	C13—C8—C9—C10	−1.5 (7)
Cl2—C2—C3—C4	179.8 (4)	C7—C8—C13—C12	179.7 (5)
S1—C2—C3—C4	−0.2 (6)	C9—C8—C13—C12	0.8 (7)
C2—C3—C4—C1	0.7 (6)	Cl3—C9—C10—C11	179.8 (4)
C2—C3—C4—C5	−179.2 (4)	C8—C9—C10—C11	1.4 (7)
C1—C4—C5—O1	169.0 (5)	C9—C10—C11—F1	−179.7 (4)
C1—C4—C5—C6	−12.0 (8)	C9—C10—C11—C12	−0.5 (8)
C3—C4—C5—O1	−11.1 (7)	F1—C11—C12—C13	179.0 (5)
C3—C4—C5—C6	167.9 (4)	C10—C11—C12—C13	−0.2 (8)
O1—C5—C6—C7	−0.5 (8)	C11—C12—C13—C8	0.0 (8)

### Hydrogen-bond geometry (Å, º)

**Table:**

*D*—H···*A*	*D*—H	H···*A*	*D*···*A*	*D*—H···*A*
C6—H6*A*···Cl1	0.93	2.47	3.207 (5)	136
C10—H10*A*···F1^i^	0.93	2.54	3.433 (6)	160

Symmetry code: (i) −*x*+3, −*y*+2, −*z*.
